# Cellular reactions in capillary and venous blood in northerners to a short‐term period in a climatic chamber

**DOI:** 10.1002/iid3.322

**Published:** 2020-06-17

**Authors:** Liliya K. Dobrodeeva, Anna V. Samodova, Svetlana N. Balashova

**Affiliations:** ^1^ N. Laverov Federal Center for Integrated Arctic Research Archangelsk Russia

**Keywords:** blood, blood cells, cold, cytokines, irisin, photoperiod

## Abstract

**Purpose:**

The purpose is a comparative study of the cellular reactions in capillary and venous blood in northerners under general hypothermia in a climatic chamber during different photoperiods. The authors examined 108 relatively healthy people (80 women and 28 men aged 21‐ to 50‐years old).

**Methods:**

The study included determining the hemogram, neutrograms, monocytograms, lymphocytograms, and phagocytic activity neutrophil granulocytes, enzyme immunoassay, flow cytometry, indirect immunoperoxidase, bioluminescence, systolic and diastolic blood pressure, and body temperature in the ear canal and on the skin of the rear left side of the right hand of volunteers before the effect of general cooling in the room at an air temperature and after 5 minutes of exposure to cold air.

**Results:**

It was established that total neutrophil count in venous blood was lower by 8.07% ± 0.41%, monocyte count by 51.32% ± 1.03%, and basophil count by 50.21% ± 1.24% than in capillary blood, but the lymphocyte count was higher by 25.23% ± 0.41% due to smaller forms that are known to be recirculating. After a 5‐minute period in a climatic chamber at −25°C in 27.53% of individuals during a polar night and in 16.51% volunteers during a polar day had elevated levels of neutrophils in the venous blood due to the increase in the levels of tumor necrosis factor‐α in blood and decrease in noradrenaline, adrenaline, and irisin.

**Conclusion:**

The systematic effect of general cooling, especially during the polar night, leads to a reduction in reserve adaptability with the formation of neutropenia, deficiency of phagocytic defense, and functional insufficiency of T‐lymphocytes.

## Key points

1. In the venous blood, the content of neutrophils, monocytes, and basophils is lower.
2. After cooling, the neutrophil content, TNF‐α, increases.
3. The effect of cooling depends on the initial content of immunocompetent cells.
4. In the polar night, a reduction in reserve adaptation possibilities was established.

**Abbreviations**
ATP: adenosine triphosphate
CD10+: precursors of B‐lymphocytes
CD16+: natural killers
CD25+: T‐lymphocytes with receptor for interleukin‐2
CD3+: mature T‐lymphocytes
CD4+: T‐helpers
CD71+: T‐cells with a receptor for transferrin
CD8+: cytotoxic T‐lymphocytes
CD95+: lymphocytes prepared for apoptosis
GM‐CSF: granulocyte–macrophage colony‐stimulating factor
HLADR II: T‐lymphocytes activated through receptors to the sublocus of the main histocompatibility complex class II
IFN: interferon
IL: interleukin
TNF‐α: tumor necrosis factor‐α

## 1 INTRODUCTION

Nearly all known studies on the composition of blood investigated venous blood. Functional activity of blood cells is observed primarily in tissues. There are data that venous and capillary blood are not identical in the rate of circulation, coagulation, erythrocyte aggregation, and rheological properties.^1^, ^2^, ^3^ The data on the leucocyte pool in capillary and venous blood are sparse and controversial. The studies by Migacheva^4^ showed that the level of leucocytes was lower and the level of erythrocytes, thrombocytes, and hemoglobin was higher in venous blood than in capillary blood. On the contrary, the study by Ledyankina et al^5^ showed that the level of leukocytes and neutrophils was 8% higher, platelets—9% higher, and monocytes—12% higher in venous blood.^5^ Despite numerous studies and published data, the issue on the different content of leucocytes and their certain forms in capillary and venous blood is not solved, and the majority of researchers believe that these differences within the physiological norm are insignificant.

The most expressed differences are observed at low temperatures of the skin.^6^, ^7^ Local hypothermia leads to the degranulation of tissue basophils and mediator‐induced edema of derma, which results in the infiltration of the tissue with mononuclear cells, neutrophils, eosinophils, and damage of endothelium with the accumulation of the immune complexes.^8^ The first reaction to general hypothermia includes a wide range of changes in general hemodynamics: the transformation of microcirculatory bloodstream with an increase in the tonus of precapillary arterioles and a decrease in the activity of the venous blood output, increase in the tonus of magistral vessels, and linear rate of the bloodstream. The data on the influence of general hypothermia on the contractility of the myocardium and parameters of cardiac output that maintain central circulation are controversial—from the maintenance of high systemic blood pressure, heart rate, and minute cardiac output to the reduction of the minute cardiac output and decrease in the heart rate. All these changes depend on the duration of the cold factor impact or adaptation of a human to cold conditions, probably, due to individual sensitivity to hypothermia. It is known that a dynamic equilibrium is established between circulating and parietal cells, which is constantly shifting to the increase or decrease of its compounds.^9^, ^10^, ^11^

Chemotactic signals and specific intercellular interactions that can be disturbed in hypothermic conditions play the main role in the determination of the migration direction and overcoming of the barriers between the blood and tissues. The immune response to general hypothermia includes a decrease in the phagocytic activity of neutrophils and monocytes in blood and the levels of activated cells and antibody production. Under the influence of cold conditions and in the winter period, qualitative and quantitative parameters of the cellular immunity are characterized by a decrease in T‐helpers and T‐suppressors by 10 to 15% and a general decrease in the functional activity of T‐lymphocytes.^12^, ^13^, ^14^ Urgent systemic adaptation of a human to any unfavorable impact includes a reaction of vasomotor amines, catecholamines, that provide the alterations in the vascular permeability, activation of the system of blood circulation, heart rate, and vascular tonus.

An extreme factor for people who live in the north is the expressed photoperiodicity. The pick of biological darkness is observed in December and January. November and February are considered biological dusk and the excess of visible light (polar day) is observed in June and July. The rhythm of physiological functions associated with the change in the regimen of day and night alters during the period of light insufficiency: the rate of metabolism, the activity of the thermoregulation, breathing, blood circulation, and higher nervous activity. The lack of light excludes a natural pathway for vitamin D, which leads to the disturbances of phosphor–calcium metabolism. In the north of the Russian Federation, a sharp change of the climatic parameters is observed during 292 days every year. High repeatability (up to 316 days every year) of discomfort types of weather is observed. A heat discomfort with a tension of mechanisms of thermoregulation is observed nearly all around the year, which leads to a significant decrease in the labor productivity up to 200% and an increase in the morbidity rate. Sharp fluctuations of temperature and photoperiodicity increase the energy consumption necessary for the maintenance of homeostasis, which reduces its reserves and periods of active performance.^13^, ^15^

The aim of the present study was to perform a comparative study of the cellular reactions in the capillary and venous blood in the northerners under general hypothermia in a climatic chamber. The authors suggested that short‐term general hypothermia can alter the perfusion of myeloid cells in tissues and recirculation of lymphocytes by the enhancement of the production of cytokines and/or vasomotor amines. The practical value of the study is in the application of the criteria of individual sensitivity of a human to general hypothermia.

## 2 MATERIALS AND METHODS

The study included 108 relatively healthy people who lived in Archangelsk (80 women and 28 men aged 21‐ to 50‐years old). During the minimum photoperiod (January and February), 63 volunteers were examined and 45 volunteers—during a polar day.

All the participants signed the informed consent form. The study was performed according to the requirements of the Helsinki Declaration (2000).

The study included the determination of systolic (BPsyst) and diastolic blood pressure (BPdiast; mm Hg), and the body temperature in the ear canal and on the skin of the back of the right hand before the effect of general cooling in the room at an air temperature of +21.3°C and after 5‐minute exposure to cold air at a temperature of −25°C in the morning from 8.00 to 10.00 in the morning.

The body temperature in the ear canal and on the skin of the back of the right hand was determined using a DT‐635 (A&D Company Ltd) medical electronic infrared thermometer (Japan) before entering and after leaving the chamber. The thermometer was installed in the ear canal and on the skin of the arm perpendicular to the surface of the body.

The volunteers dressed in underwear, cotton outerwear, and replaceable shoes were in a climate chamber in a standing position at rest.

The authors took samples of venous and capillary blood from the IV finger of the examined volunteers between 8.00 and 10.00 in the morning on an empty stomach before and after 5‐minute hypothermia at −25°С in a climatic chamber (USHZ‐25N, Russia) during a minimum duration of a photoperiod (January) and polar day (June).

In venous and capillary blood, the authors identified hemogram with an automatic hematologic analyzer XS‐500i (Sysmex, Japan) and in blood smears that were Romanowsky–Giemsa stained. Neutrogram was calculated by the method of Todorov (1968), monocytorgam by the method of Grigorova (1956), lymphocytogram by the method Kassirskiy (1970), and phagocytic activity of neutrophil granulocytes was estimated in peripheral blood. The concentration of irisin, endothelin‐1, cytokine tumor necrosis factor‐α (TNF‐α), adrenaline, and noradrenaline in blood was identified by the method of enzyme‐linked immunoassay with an automatic immunoenzymometric analyzer Evolis (Bio‐Rad, France); the content of lymphocyte phenotypes (CD3+, CD4+, CD8+, CD10+, CD16+, CD23+, CD25+, CD71+, CD95+, HLA DR+)—with an indirect immune peroxidase reaction with monoclonal antibodies (MedBioSpectr; Sorbent, Moscow, Russia), and the method of flow cytometry with an apparatus Epics XL (Beckman Coulter), chemical reagents—Immunotech, a Beckman Coulter Company (France). The changes in the levels of adenosine triphosphate (ATP) in lymphocytes were evaluated with a luminometer LUM‐1 (Lumtech, Russia) and a standard chemical reagent kit Lumtech.

Mathematical analysis of the obtained results was performed with Microsoft Excel 2010 and Statistica 7.0 (StatSoft). The distribution of the values of immunological parameters was checked with the Pearson test. Null hypothesis on the equality of all the means in the studied groups was checked with a one‐factor dispersion analysis. When the distribution was abnormal, the comparison of two different groups by quantitative parameters was performed by nonparametric Mann–Whitney test. The authors calculated the parameters of the descriptive statistics (M: mean arithmetic, σ: standard deviation, m: standard error of the mean, Md: median, R: range, W: variation coefficient, 95% confidence interval). A critical level of significance (*P*) was taken as .05.

## 3 RESULTS

After a short stay in the cold chamber for 5 minutes at a temperature of −25°C in the examined individuals, the temperature of the skin of the hand decreases from 34.28 ± 0.28 to 23.78°C ± 0.62°C (*P* < .001) without significant differences in body temperature and in the ear canal (36.80 ± 0.08 and 36.00°C ± 0.80°C; *P* = .356). BPsyst and BPdiast do not significantly change under these conditions (123.88 ± 1.38 and 125.40 ± 1.44; *P* = .096; 75.78 ± 0.98 and 76.35 ± 0.987mm Hg, respectively; *P* = .477).

A comparative analysis of the cellular composition of venous and capillary blood in the examined volunteers before the climatic chamber showed that venous blood had lower content of neutrophils by 8.07% ± 0.41%, primarily due to segmentonuclear cells with two to three segments of nuclei from 1.02 ± 0.06 to 0.85 ± 0.05 × 10^9^ cells/L and from 1.34 ± 0.06 to 1.24 ± 0.06 × 10^9^ cells/L, respectively. The concentration of monocytes in venous blood decreased by 51.32% ± 1.03% due to promonocytes (from 0.34 ± 0.04 to 0.16 ± 0.02 × 10^9^ cells/L), mature monocytes (from 0.30 ± 0.02 to 0.16 ± 0.01 × 10^9^ cells/L), and polymorphonuclear cells (from 0.12 ± 0.01 to 0.05 ± 0.004 × 10^9^ cells/L). A decrease in basophils by 50.21% ± 1.24% was also registered. The content of lymphocytes in venous blood was significantly higher (by 25.32% ± 0.41%), including their smaller forms (from 1.10 ± 0.06 to 1.42 ± 0.08 × 10^9^ cells/L) that are known to be recirculating (Table 1).

**Table 1:** Comparison data of the cellular composition of venous and capillary blood in northerners before a climatic chamber session (M ± m)

Cells, 10^9^ cells/L	Capillary blood	Venous blood
1	2	3
Leukocytes	5.88 ± 0.19	5.80 ± 0.19
Neutrophils	3.22 ± 0.13	2.96 ± 0.14*
Banded neutrophile	0.16 ± 0.01	0.23 ± 0.02***
Segmentonuclear neutrophils	3.06 ± 0.01	2.73 ± 0.13***
Monocytes	0.76 ± 0.06	0.37 ± 0.03***
Eosinophils	0.11 ± 0.01	0.12 ± 0.01
Basophiles	0.04 ± 0.01	0.02 ± 0.003**
Lymphocytes	1.74 ± 0.07	2.33 ± 0.11***

*Note*: During a comparison of capillary and venous blood in the examined patients

* *P* < .05.

** *P* < .01.

*** *P* < .001.

The authors suggest that the decrease in the content of neutrophils, monocytes, and basophils in venous blood in comparison with capillary blood was observed due to perfusion of cells to tissues, and the increase in the content of lymphocytes in venous blood was observed due to a possible recirculation of lymphocytes. The lack of a clear difference in monocytograms of capillary and venous blood could indicate the ability of monocytes to recirculate.

After a 5‐minute hypothermia session in a climatic chamber at −25°С with 25 (27.53%) volunteers during a polar night and 16 (16.51%) volunteers during a polar day, an increased content of neutrophils in capillary and venous blood was observed from 3.41 ± 0.20 to 4.26 ± 0.35 × 10^9^ cells/L (*P* < .001) and from 2.70 ± 0.17 to 3.02 ± 0.22 × 10^9^ cells/L (*P* < .001), including segmentonuclear cells from 3.25 ± 0.25 to 4.11 ± 0.33 × 10^9^ cells/L (*P* < .001) and from 2.46 ± 0.16 to 2.76 ± 0.21 × 10^9^ cells/L (*P* < .001). No changes were observed in the content of eosinophils (0.11 ± 0.02 and 0.10 ± 0.02 × 10^9^ cells/L and 0.13 ± 0.02 and 0.11 ± 0.01 × 10^9^ cells/L) and basophils (0.01 ± 0.001 and 0.01 ± 0.001 × 10^9^ cells/L and 0.02 ± 0.01 and 0.03 ± 0.01 × 10^9^ cells/L). Thus, the direction of the reactions of the changes in the content of neutrophils, eosinophils, and basophils to hypothermia in summer and winter was similar—under the influence of hypothermia, the activity of the perfusion of myeloid cells in tissues decreased. A decrease in the level of lymphocyte recirculation from 1.76 ± 0.13 to 1.40 ± 0.11 × 10^9^ cells/L (*P* < .01) and from 1.95 ± 0.11 to 1.65 ± 0.10 × 10^9^ cells/L (*P* < .01), primarily of smaller forms from 1.19 ± 0.10 to 1.00 ± 0.09 × 10^9^ cells/L (*P* < .01) and from 1.55 ± 0.11 to 1.18 ± 0.08 × 10^9^ cells/L (*P* < .001), was registered only during a polar night.

One of the objectives was to reveal if there were changes in the functional activity of lymphocytes in venous blood after a session in a climatic chamber (Table 2). The obtained data showed that the decrease in the recirculation of lymphocytes was associated with a significant decrease in the functional activity of all the studied phenotypes of lymphocytes.

**Table 2:** Content of lymphocyte phenotypes in the peripheral venous blood in patients that react to general hypothermia before and after climatic chamber during a polar night (М ± m)

Parameters, 10^9^ cells/L	Before a session in a climatic chamber	After a session in a climatic chamber
Lymphocytes	1.95 ± 0.11	1.65 ± 0.10**
CD3+	0.90 ± 0.04	0.40 ± 0.02***
CD25+	0.40 ± 0.03	0.24 ± 0.02***
CD71+	0.40 ± 0.03	0.22 ± 0.02***
HLADRII	0.39 ± 0.03	0.24 ± 0.02***
CD10+	0.36 ± 0.03	0.18 ± 0.02***
CD4+	0.44 ± 0.04	0.20 ± 0.02***
CD8+	0.39 ± 0.04	0.17 ± 0.01***
CD16+	0.38 ± 0.03	0.16 ± 0.02***
CD23+	0.33 ± 0.03	0.15 ± 0.02***
CD95+	0.28 ± 0.02	0.14 ± 0.02***

*Note*: During a comparison of capillary and venous blood in the examined patients

** *P* < .01.

*** *P* < .001.

Volunteers that reacted to a short‐term hypothermia, before a session in a climatic chamber, had higher content of neutrophils; their phagocytic activity and the concentration of lymphocytes (2.46 ± 0.13 and 1.70 ± 0.15 × 10^9^ cells/L; *P* < .001), including T‐cells with receptors to transferrin (0.40 ± 0.03 and 0.29 ± 0.02 × 10^9^ cells/L; *P* < .01), was higher. These volunteers had higher content of ATP in lymphocytes (2.48 ± 0.17 and 0.88 ± 0.13 µmol/mln cells; *P* < .001), TNF‐α (2.12 ± 0.34 and 1.23 ± 0.27 pg/mL; *P* < .001), irisin (5.60 ± 0.70 and 3.74 ± 0.55 µg/mL; *P* < .01), and adrenaline (53.03 ± 7.29 and 33.27 ± 4.07 pg/mL; *P* < .01) in blood serum without significant changes in the content of endothelin‐1 (0.75 ± 0.11 and 0.77 ± 0.18 fmol/mL; Table 3).

**Table 3:** Content of TNF‐α, adrenaline, and short peptides in the peripheral venous blood in patients that react to general hypothermia before and after climatic chamber during a polar night (М ± m)

Parameters	Before a session in a climatic chamber	After a session in a climatic chamber
TNF‐α, pg/mL	2.12 ± 0.34	1,23 ± 0.27***
Adrenalin, pg/mL	53.03 ± 7.29	33,27 ± 4.07**
Irisin, µg/mL	5.60 ± 0.70	3,74 ± 0.55**
Endothelin‐1, fmol/mL	0.75 ± 0.11	0,77 ± 0.18

*Note*: During a comparison of capillary and venous blood in the examined patients

Abbreviation: TNF‐α, tumor necrosis factor‐α

** *P* < .01.

*** *P* < .001.

## 4 DISCUSSION

Functional activity of blood cells was observed primarily in tissues, where the content of monocytes was 51.32% ± 1.03%, basophils—50.21% ± 1.24%, and neutrophil granulocytes—8.07% ± 0.41%. The concentration of lymphocytes in venous blood was higher than in capillary by 25.32% ± 0.41% due to smaller forms that are believed to represent the majority of recirculating cells. Recirculating lymphocytes are small, primarily T‐cells of the reserve pool, that are capable of further blast‐cell transformation and differentiation.^16^, ^17^, ^18^, ^19^, ^20^

The change in the ratio of circulating and parietal pools is the main signal for hemodynamic reaction. Microvasculature is a system of transport bloodstream, its functional condition changes depending on the status of tissues that provide these areas with blood.^21^, ^22^, ^23^, ^24^, ^25^, ^26^, ^27^, ^28^ Functional condition of the microvasculature is provided by numerous regulatory mechanisms of endothelial origin with secretion of nitrogen oxide and vasoconstrictor endothelin‐1, neurogenic sympathetic activity, myogenic mechanisms (peptide ergic and endogenous), as well as pulse and respiratory oscillations. The fastest mechanism of regulation of microvasculature is endothelial. The deficit of endothelial‐dependent vasodilation is caused by a shift in the balance of the synthesis of nitrogen oxide and vasoconstrictors to the domination of vasoconstrictors, primarily, endothelin‐1.^29^, ^30^ The main depo of neutrophils is located in the capillary net of lungs. Lungs have nearly unlimited depositing capacity reserving primarily active granulocytes.^31^ Lymphocytes are different by their capability to recirculation. It is suggested that monocytes are capable of recirculation; the tissue pool of monocytes exceeds the content of tissue neutrophils by 3.5 times.^32^

Migration and perfusion of cells are provided by a significant reduction of the bloodstream rate in the capillary net, which is observed in hypothermic conditions. A cell sticks to the capillary walls with its further migration outside the vascular net.^16^, ^17^, ^20^ More than that, molecules of adhesion provide a selection of cells that stick to the capillary walls. In problem areas, adhesive molecules of endothelium bind leukocytes and transmit a signal required for the transendothelial migration, which is regulated by the interaction of CD47 and signal regulatory protein of leukocytes. Binding of ligands with CD47 initiates signaling from endothelial cells, which contributes to transendothelial migration.^33^ A change in the activity of endothelial cells under the influence of inflammatory cytokines is accompanied by a formation of gaps and an increase in the permeability of cells.^34^

Hypothermia causes degranulation of tissue basophils and edema of the derma due to the effect of mediators, which leads to the infiltration of the tissue with mononuclear cells, neutrophils, eosinophils, and the damage of endothelium with the accumulation of the immune complexes.^8^ The products of lipid peroxidation, that are actively involved in numerous metabolic processes, affect vascular endothelial cells causing vasoconstriction and damaging cellular and subcellular membranes, which leads to disturbances in the processes of capillary trophic and gas exchange.^35^, ^36^ A number of authors report that local hypothermia leads to the increase in the level of catecholamines, corticosteroids, and histamines.^37^, ^38^, ^39^, ^40^

In the majority of the volunteers, short‐term hypothermia did not influence the content of leukocytes, the activity of their migration to tissues, and the level of recirculation of lymphocytes. The reaction of inhibition of migration activity to general hypothermia was registered in 20.52% of volunteers with a significant increase in the rate of reactions in winter (27.53% and 16.51%, respectively). It was established that people with a reaction of inhibition of migration activity of leukocytes had some background peculiarities in the performance of the studied parameters. Volunteers that reacted to short‐term hypothermia, had a higher content of neutrophils, their phagocytic activity, concentration of lymphocytes, including T‐cells with a receptor to transferrin, before a session in a climatic chamber. These volunteers had a higher content of ATP in lymphocytes, TNF‐α, irisin, and adrenaline in blood serum without significant changes in the concentration of endothelin‐1. Increase in the energy resources can be provided by the increase in the production of TNF‐α and irisin, as well as additional transport of iron to cells. TNF‐α plays an important role during the first minutes of the development of the inflammatory reaction because it activates endothelium and contributes to the expression of adhesive molecules, which makes granulocytes stick to the internal surface of a vessel. Under the influence of TNF‐α, transendothelial migration of leukocytes to the focus of inflammation is observed. This cytokine activates granulocytes, monocytes, and lymphocytes, and induces the production of other anti‐inflammatory cytokines interleukin‐1 (IL‐1), IL‐6, interferon, and granulocyte‐macrophage colony‐stimulating factor which act as synergists of TNF‐α.^41^ There are data that confirm the stimulation of recirculation of lymphocytes with the present cytokine.^13^

It is known that adrenaline enhances tissue exchange, and inhibits the expression of histamine, serotonin, and kinins, which can inhibit the perfusion of cells via membranes. Probably, an increase in the level of adrenaline and a lack of the reaction of endothelin‐1 are the main stages in a chain of reactive processes that regulate the reactions of migration of leukocytes to hypothermia.

## 5 CONCLUSION

Thus, it is suggested that the mechanisms of maintaining homeostasis under conditions of general cooling are based on the principle of modulation regulation of the activity of various systems, which provides the optimal stability option in changing environmental conditions, based on the background possibilities of regulation. Increased production of TNF‐α, irisin, and ATP, the functional activity of neutrophil granulocytes, and T‐lymphocytes stimulate cell migration into the tissue and lymphocyte recirculation. The expression of the pre‐endothelin‐1 and proendothelin‐1 genes and the release of the active peptide are stimulated by various humoral factors (angiotensin II, IL‐1, adrenaline, norepinephrine, vasopressin, and thrombin), including TNF‐α and ATP. Endothelin is one of the most potent vasoconstrictors. Apart from the vasoconstrictive effect, it enhances the production of cytokines.^42^, ^43^ The ambiguity of the response of tissue blood flow to the effect of cooling is predetermined by the different initial state of functioning of the microvasculature. The systematic effect of general cooling, especially during the polar night (heat deficiency), leads to a reduction in reserve adaptability with the formation of neutropenia, deficiency of phagocytic protection, and functional insufficiency of T‐lymphocytes.

## CONFLICT OF INTERESTS

The authors declare that there are no conflict of interests.

## Supporting information

Supporting information
