# Supplementary material for: Cellular reactions in capillary and venous blood in northerners to a short‐term period in a climatic chamber
Source: Immun Inflamm Dis. 2020 Jun 17;8(3):408–14. doi: 10.1002/iid3.322 (PMC7416046; doi:10.1002/iid3.322)
Supplement: Supplementary file 1 — Supporting information [file IID3-8-408-s001.docx]

1. The protocol of the study.

2. Description of the scientific research.

3. Form of voluntary informed approval of the subject.

4. Information for the subject.

5. Documents confirming the qualification of the researcher (copies of the diploma)

6. Applications.

7. Consent to the processing of personal data.

8. Attached documents:

Researcher contact details: tel.: + 7 (962) 6647328;

  e-mail: (hidden) [annapoletaeva2008@yandex.ru](mailto:annapoletaeva2008@yandex.ru).

___________ Signature of researcher

**THE STUDY PROTOCOL**

**General data**

1. Name of researcher: Samodova Anna Vasilievna.

Position: leading researcher, head of laboratory of regulatory mechanisms in immunity, Institute of environmental physiology, INSTITUTE of VICKIE wounds.

2. Date of report: 25.01.2017

3. The title of the study: "the Role of the extracellular pool of adhesion molecules and short peptides in the formation and outcome of adaptive reactions to changing light conditions"

4. Supervisor of studies: candidate of biological science., head. laboratory of regulatory mechanisms in immunity Samodova Anna Vasilievna.

5. Proof of qualification of the researcher:

The status of a researcher – candidate of biological Sciences.

Work experience – 12 years

The total number of works – 88.

Diploma, certificate diploma of higher education, diploma of candidate of biological Sciences.

6. Insurance researcher at the risks associated with the conduct of this study (underline).

- is provided.

- not applicable.

7. Place of study: Institute of physiology of natural adaptations, Arkhangelsk.

8. Whether the study is multicenter: no.

9. The organization initiating the study: the Institute of environmental physiology, INSTITUTE of VICKIE wounds.

10. Funding source research:

- the state budget;

- funds received through grants;

11. Description of actions to stakeholders to participate in the study: all results of immunological examination of study subjects.

**DESCRIPTION RESEARCH**

1. The aim of the study was to establish the role of the quantitative content of the extracellular pool of receptors and the level of short peptides (brain natriuretic peptide, endothelin-1, irisin) in the regulation of immune responses in human adaptation to cold depending on the change of light conditions (polar night and polar day).

2. Planned research terms: 2017-2022.

3. The number and type of patients/ healthy volunteers (outpatient, inpatient, etc.): 150 healthy volunteers.

4. The age of the subjects (upper/lower bounds): 18 – 60 years.

5. The presence of vulnerable groups of subjects were unemployed, low-income:

- Yes;

- no.

6. Inclusion criteria in the study group: healthy men and women aged 18 to 60 years residing in the city of Arkhangelsk.

7. Exclusion criteria: the presence of chronic diseases or complaints of malaise, affecting working capacity.

8. The number and type of experimental animal: no.

9. Long duration of the study for one subject: about 10-15 min.

10. Randomization procedure – provided, not provided.

11. The nature of the study:

11.1. experimental in laboratory animals;

11.2. diagnostic;

11.3. pharmacological;

11.4. therapeutic;

11.5. social-hygienic;

11.6. other;

12. The list of drugs whose effect the researcher is studying (including placebo): no.

13. The list of medical products used in the study:

13.1. allowed to use (unabridged): test tubes for blood sampling Kometaline: tubes with clotting activator (SiO2); test tube with EDTA K2/K3, medical gloves, skin antiseptic, "Absolutepath", apparatus for blood pressure measuring, scales, stadiometer;

13.2. new, announced for registration in the prescribed legal manner: not available;

13.3. approved for use, but are the new readings: no.

**ASSESSMENT OF RATIO OF BENEFIT AND RISK RESEARCH**

1. The use of the proposed study:

1.1. the study serves directly the interests of the subjects: Yes;

The complex immunological studies, including a study of the haemogram, phagocytic activity of neutrophils, lymphocyte phenotypes, determination of the level of concentration of immunoglobulins, CEC, cytokines, short peptides, membrane-free forms of antigens in the serum allows you to fully assess the immune system status of research subjects.

1.2. the study is obtaining important results aimed at improving methods of diagnostics, prevention and treatment: Yes;

2. This scientific study:

2.1. does not go beyond the conventional diagnostic-therapeutic complex is used in these cohorts of patients.

3. Risk research:

3.1. there is no risk;

3.2. the risk is minimal;

3.3. the risk is more than minimal.

4. Risk:

4.1. with the use of diagnostic procedures (blood sampling);

4.2. with the use of medicines (specify what);

4.3. with the use of surgical methods of treatment (specify what)

4.4. more

Signature of researcher

**FORM VOLUNTARY INFORMED CONSENT OF THE SUBJECT**

Informed consent for examination

Home address

Contact phone

I, (name) years

give informed consent for participation in scientific research, in the cold chamber at the temperature -25° C, held at the initiative of laboratory of ecological immunology and regulatory mechanisms of immunity examination, which I was explained in detail during the inspection. I was(a) informed(a) about the goals and objectives of the present study. I know that I should(a) will answer the questions in the questionnaire about my social status, nutrition, health, habits, physical activity, and undergo anthropometric measurements: height, weight, waist circumference, hip circumference, blood pressure measurements and heart rate.

I also understand that I will need(a) to take blood from a vein. Blood will be taken by medical personnel sterile.

I understand that the results will be reviewed with strict confidentiality.

I understand that the results of this research work may be published in medical and scientific journals; however, my name and my family name will not be mentioned. The results will be presented in the group of participants.

I understand that my participation is voluntary and I can withdraw from the project at any time without notice. I also understand that if I ask, the results will be available to me.

Date ________________________

Signature ____________________

I hereby Express my consent to the processing of personal data by the laboratory of biological and inorganic chemistry, Institute of environmental physiology, INSTITUTE of VICKIE wounds. Confirm that you have read(a) with the provisions of the Federal law of 27.07.2006 №152ФЗ "ON personal data". Rights and obligations in the field of personal data protection I understand.

**INFORMATION FOR THE PATIENT**

1. The purpose of this study is to establish the role of the quantitative content of the extracellular pool of receptors and the level of short peptides (brain natriuretic peptide, endothelin-1, irisin) in the regulation of immune responses in human adaptation to cold depending on the change of light conditions (polar night and polar day).

2. As subjects of the study examines men and women aged 18 to 60 years residing in the city of Arkhangelsk.

3. Examination will be held simultaneously in the morning on an empty stomach, the blood will be taken from the cubital vein. Will be pre-surveyed.

4. Procedures performed in the course of this study, have minimal risk to health.

5. The decision to participate in this study must be accepted by You voluntarily and will not affect Your future professional career.

6. Before You decide to participate in the study, You have the right to obtain any information about the study.

7. All study-related medical records and research materials that could identify You will be kept confidential and will not be publicized. In case of publication of study results in the literature, Your identity will not be revealed and cannot be identified.

8. At the time of the study insurance risks associated with Your participation in the study is not provided.

9. With the development of adverse reactions, complications related to the implementation of screening, treatment, You will be provided with qualified medical assistance in accordance with the contract of obligatory medical insurance.

10. Contact information: Samodova Anna Vasilievna, candidate of biological Sciences, head of laboratory of regulatory mechanisms in immunity, Institute of environmental physiology, INSTITUTE of VICKIE ran: Arkhangelsk, Avenue of Lomonosov, d. 249. Phone: +7(962)6647328.

FEDERAL STATE BUDGETARY INSTITUTION OF SCIENCE

Institute of ecological physiology INSTITUTE of RAS VICKIE

Entry form

Name:

Date of birth: Gender: M  W  Height: Weight:

BLOOD PRESSURE: HEART RATE:

Place of birth:

Give blood on an empty stomach? Yes  No 

Place of work:

Post:

Work experience:

How many years have You live in the North?

Do You have a chronic illness or health problems? Yes  No 

If Yes, then what you have a chronic illness:

Do You use drugs constantly/on a regular basis? Yes (please list)

No

Have You used drugs in the last 5 days? Yes (please list)

No
